# The Potential Role of Gossypetin in the Treatment of Diabetes Mellitus and Its Associated Complications: A Review

**DOI:** 10.3390/ijms242417609

**Published:** 2023-12-18

**Authors:** Karishma Naidoo, Andile Khathi

**Affiliations:** Department of Human Physiology, School of Laboratory Medicine and Medical Sciences, University of KwaZulu-Natal, Durban 4000, South Africa; 217000603@stu.ukzn.ac.za

**Keywords:** gossypetin, diabetes mellitus, flavonoid

## Abstract

Type 2 diabetes mellitus (T2DM) is a metabolic disorder caused by insulin resistance and dysfunctional beta (β)-cells in the pancreas. Hyperglycaemia is a characteristic of uncontrolled diabetes which eventually leads to fatal organ system damage. In T2DM, free radicals are continuously produced, causing extensive tissue damage and subsequent macro-and microvascular complications. The standard approach to managing T2DM is pharmacological treatment with anti-diabetic medications. However, patients’ adherence to treatment is frequently decreased by the side effects and expense of medications, which has a detrimental impact on their health outcomes. Quercetin, a flavonoid, is a one of the most potent anti-oxidants which ameliorates T2DM. Thus, there is an increased demand to investigate quercetin and its derivatives, as it is hypothesised that similar structured compounds may exhibit similar biological activity. Gossypetin is a hexahydroxylated flavonoid found in the calyx of *Hibiscus sabdariffa*. Gossypetin has a similar chemical structure to quercetin with an extra hydroxyl group. Furthermore, previous literature has elucidated that gossypetin exhibits neuroprotective, hepatoprotective, reproprotective and nephroprotective properties. The mechanisms underlying gossypetin’s therapeutic potential have been linked to its anti-oxidant, anti-inflammatory and immunomodulatory properties. Hence, this review highlights the potential role of gossypetin in the treatment of diabetes and its associated complications.

## 1. Introduction

**Figure 1:**
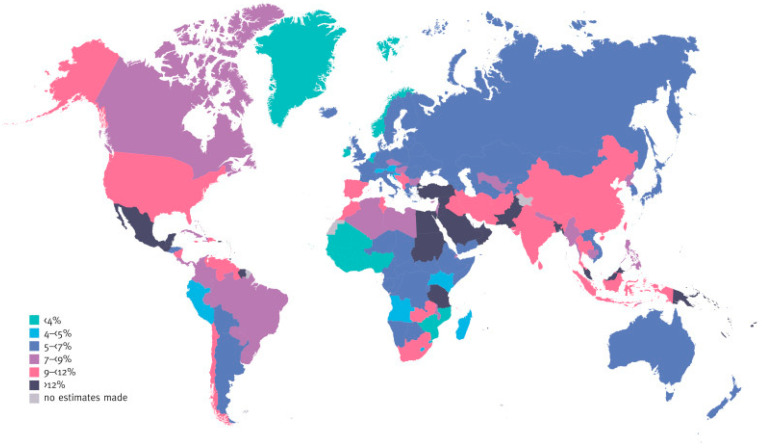
Estimated age-adjusted comparative prevalence of diabetes in adults (20–79 years) in 2021 Adapted with permission from International Diabetes Federation, 2013 [3].

Approximately 90% of all cases of diabetes are type 2 diabetes mellitus (T2DM) [1]. The global prevalence of T2DM in adults was 536.6 million in 2021, and by 2045, 783.2 million people are expected to have the condition, according to the International Diabetes Federation (IDF) (Figure 1) [2,3]. The response to insulin is diminished in T2DM, which is characterised as insulin resistance [4]. It has been demonstrated that macro- and microvascular complications such as atherosclerosis, stroke, kidney failure and non-alcoholic fatty liver disease (NAFLD) are associated with diabetes mellitus (DM) [5,6]. Oxidative stress has been demonstrated to be an essential component in the pathophysiology of these complications [7]. Unfortunately, conventional medical treatments for T2DM exhibit unfavourable side effects, such as adverse reactions in the gastrointestinal tract and damage to the kidneys and liver [8].

There has been increased global interest currently to identify anti-oxidant compounds that are pharmacologically potent with little or no side effects [9]. As the primary source of anti-oxidants, flavonoids have emerged as an effective tool to combat against oxidative stress [10]. According to previous studies, one of the most prevalent flavonols derived from plants is quercetin, which has demonstrated various anti-diabetic effects, along with reduced side effects [11,12]. Thus, due to the notable therapeutic activities of quercetin, it has gained considerable interest [11]. Moreover, there exists an extensive reserve of phytochemicals that remain unexplored but may possess similar therapeutic properties to quercetin [13,14]. It has been demonstrated that similarly structured compounds exhibit similar biological activity [13]. Moreover, research has demonstrated that the number and location of hydroxyl groups in flavonoids affect their anti-oxidant activity [15]. Gossypetin is a flavonoid that is found in the calyx of *Hibiscus sabdariffa* [16]. Interestingly, gossypetin has been shown to exhibit a similar structure to quercetin with an extra hydroxyl group, which may suggest more potent anti-oxidant activity [17]. Relevant to this review, studies have demonstrated the anti-oxidant, anti-inflammatory, nephroprotective, neuroprotective and hepatoprotective properties of gossypetin, all without any discernible toxicity [18,19,20]. Gossypetin has been demonstrated to be an effective dual-targeting agent that activates AMP-activated protein kinase (AMPK) and reduces oxidative stress, in contrast to conventional anti-diabetic medications that target one pathological mechanism [21]. In addition, the *Hibiscus* species is easily accessible and offers an inexpensive source of gossypetin [22]. The aim of the present review is to elucidate the potential role of gossypetin in the treatment of diabetes and its associated complications.

### 1.1. Type 2 Diabetes Mellitus Complications

**Figure 2:**
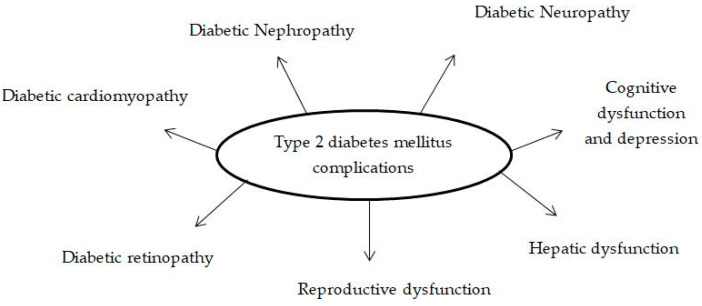
The complications associated with type 2 diabetes mellitus [5,6,27].

Type 2 diabetes (T2DM) is a chronic metabolic disease that is rapidly becoming more prevalent across the world [23]. It is characterised by insulin resistance and dysfunction in the β-cells in the pancreas [24]. The pathophysiology and progression of this metabolic disorder is associated with hyperglycaemia-induced reactive oxygen species (ROS) production and oxidative stress [7]. Oxidative stress under hyperglycaemic conditions is caused by an imbalance between the production of ROS and the cellular anti-oxidant system [7]. This leads to the development of diabetes [7]. Peroxisomes, phagocytic cells and the endoplasmic reticulum all produce reactive oxygen species (ROS), with the mitochondrial electron transport chain (ETC) playing a major role [25]. Structural and functional changes in proteins, lipids and nucleic acids have been shown to be induced by the increased production of ROS [26]. Moreover, ROS alters multiple intracellular signaling pathways, which results in insulin resistance and decreased β-cell performance [7]. In addition, the generation of ROS induced by hyperglycaemia also plays a role in the development of the macro- and microvascular complications associated with diabetes (Figure 2) [5,6,27]. Furthermore, it has been suggested that the generation of ROS through hyperglycaemia triggers several stress-sensitive signaling pathways, such as p-38 mitogen-activated protein kinases (MAPK), nuclear factor kappa B (NF-κB) and Jun amino-terminal kinases/stress-activated protein kinases (JNK/SAPK), which in turn accelerates the development of complications related to T2DM [27]. Furthermore, research has demonstrated that the development of microvascular diabetic complications is significantly influenced by pro-inflammatory cytokines [28,29]. Thus, clinical treatments may be developed from therapeutic approaches based on anti-oxidant and anti-inflammatory properties that have beneficial action against diabetic complications [30].

### 1.2. Flavonoids

**Figure 3:**
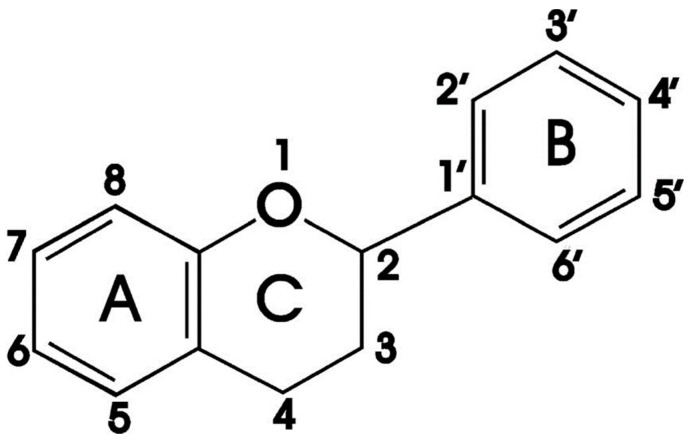
General structure of flavonoids. Adapted with permission from Ekalu and Habila,2020 [34].

The majority of phytochemicals with anti-oxidant activity in plants are phenolics, which make up the largest class of phytochemicals [31]. Of all the phenolic compounds that occur naturally, flavonoids make up the largest group [32]. They are responsible for the different colours seen in leaves, seeds, bark, flowers and fruits [10]. The fundamental flavone skeleton of flavonoids is composed of 15 carbons and includes two benzene rings (A and B) connected by a three-carbon pyran ring (C) (Figure 3) [33,34]. The distinct subclasses of flavonoidal compounds are derived from variations in this fundamental structure (Figure 3) [10,34]. These include chalcones, anthocyanins, flavanones, isoflavones, flavones and flavanols [10]. These secondary metabolites have been the subject of recent research due to their potential to prevent metabolic disorders such as obesity and diabetes mellitus [35]. The research findings indicate that dietary flavonoids have the potential to enhance insulin sensitivity, improve dysregulated lipid metabolism and reduce oxidative stress in metabolic disorders [36]. The research has shown that quercetin, one of the most abundant flavonols found in plants, has potent anti-diabetic properties [37,38].

#### 1.2.1. Quercetin

**Figure 4:**
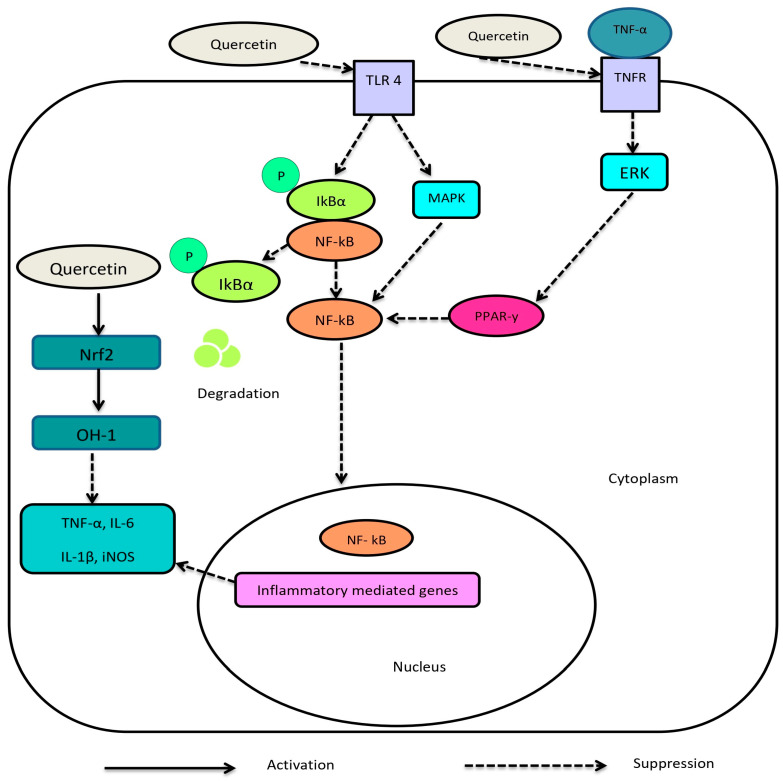
The potential molecular mechanism of quercetin on the NF-kB inflammatory pathway [44,45]. The activation of toll-like receptor (TLR) pathways encourages the production of pro-inflammatory cytokines through the upregulation of transcription factors such as NF-κB [44,45]. Quercetin suppresses inflammatory cytokines production such as interleukin (IL)-6, tumour necrosis factor (TNF)-α and IL-1β via downregulating TLR4 [46,53]. Subsequently, repressed receptors yield targeted suppression of IκBα phosphorylation and mitogen-activated protein kinase (MAPK) expression and thereby inhibit nuclear transfer of the NF-κB [46,47]. Phosphorylation is represented as p in the above figure. In addition, quercetin may indirectly prevent inflammation by increasing peroxisome proliferator-activated receptor c (PPARγ) activity, thereby antagonising NF-κB [46,48]. In addition, TNF-α binds to TNF receptors, which causes the activation of extracellular signal-regulated kinase (ERK) [46]. Activated ERK inactivates PPARγ, therefore preventing nuclear translocation and the DNA binding of NF-κB [46,54]. This prevents the transcription of inflammatory mediators and triggers oxidative stress [46]. Quercetin activates nuclear factor erythroid 2-related factor 2 (Nrf2) and thereby contributes to increased heme oxygenase (HO)-1 levels, which are responsible for the downregulation of TNF-α [46]. Together, these block NF-kB-mediated induction of inflammatory cascades [46].

Quercetin is a flavonoid that is mainly found in citrus fruits, grapes, berries and broccoli [11]. It exhibits potent anti-oxidant properties [39]. Studies have indicated that quercetin is a beneficial natural substance that regulates important signaling pathways and acts on various diabetes targets [40]. Unlike the currently used therapeutics, quercetin improves both hyperglycaemia and its associated macrovascular and microvascular complications [12,37]. Quercetin increases the production of insulin, protects pancreatic beta-cells from oxidative stress and improves the cells’ ability to defend against reactive oxygen species [37,41]. Diabetic complications including retinopathy, nephropathy and neuropathy have been shown to be induced by inflammation and oxidative stress [42,43]. Quercetin has been shown to prevent diabetic complications by blocking NF-κB cells, monocyte chemoattractant protein 1 (MCP-1) and intercellular adhesion molecule 1 (ICAM1) in T2DM patients (Figure 4) [44,45,46,47,48]. Moreover, quercetin has been shown to decrease nephropathy biomarker levels, such as creatinine, blood urea nitrogen (BUN) and 8-hydroxydeoxyguanosin [49,50]. Furthermore, the research has demonstrated that quercetin has a safer profile than conventional anti-diabetic medications that are marketed [37,51]. Additionally, a novel fermentation-based glycosylation technique using inexpensive substrates such as underutilised food waste can be used to produce quercetin on a large scale [37]. Furthermore, a study showed that similar structured compounds may exhibit similar biological activities [13]. There exists a large pool of unexplored compounds with similar therapeutic activities to quercetin [52].

#### 1.2.2. Gossypetin

**Figure 5:**
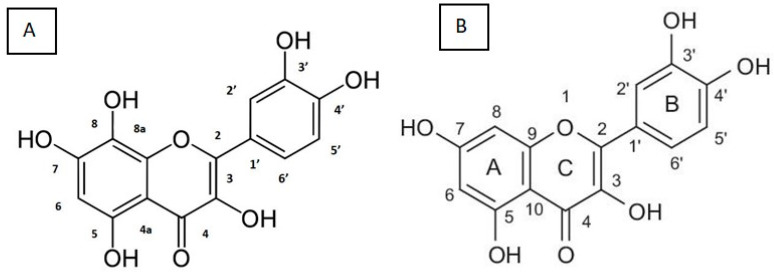
Structure of (**A**) gossypetin (GTIN) and (**B**) quercetin. Adapted with permission from Khan et al., 2013 and Zheng and Chow, 2009 [55,63].

Gossypetin (GTIN) is a natural derivative of well-known quercetin [55]. It has previously been noted that quercetin is beneficial against diabetes mellitus and its associated complications [21,37]. Gossypetin is hexahydroxyflavone and is found in the calyx of *Hibiscus sabdariffa* [16]. Tropical regions have made extensive use of these calyces as potential sources of medicinal phytochemicals [56]. Traditionally, these plant extracts were used to treat inflammation, jaundice and diabetes [57,58]. GTIN is a yellow pigment, with a chemical formula of C15H10O8, a molecular weight of 318.24 g/mol and a boiling point temperature of 679.30 °C @ 760.00 mmHg [59]. Since reducing agents exhibit anti-oxidant properties, an organic molecule’s reducing power may serve as an indicator to evaluate its anti-oxidant activity [60]. The anti-oxidant actions of flavonoids are mediated by their functional hydroxyl groups, which chelate metal ions and scavenge free radicals [61]. The number and arrangement of hydroxyl groups in flavonoids also significantly influence their anti-oxidant properties [15]. The high anti-oxidant activity which gossypetin exhibits may be attributed to the presence of a carbonyl group at position C4, a double bond between C2 and C3 and the 3-OH and 4-OH groups in the B ring (Figure 5) [55,62,63]. Interestingly, gossypetin has a catechol moiety as a B-ring, similarly to quercetin [17]. In addition, gossypetin has three hydroxyl groups rather than two at positions 5, 7 and 8 in the A-ring (Figure 5) [17,55,63]. In gossypetin, the two subsequent hydroxyl groups at positions 7 and 8 function as another catechol-like center [17]. This suggests that gossypetin has three oxidisable redox active centers while quercetin only has two [17]. In addition, previous research showed that among 15 well-known flavonoids, including quercetin, gossypetin possessed the most potent reducing capacity [17]. This may suggest that gossypetin exhibits more potent anti-oxidant activity than quercetin. In addition, gossypetin may exhibit similar pharmacological properties to quercetin due to the structural similarity. Studies have shown that gossypetin exerts various pharmacological properties, including anti-oxidant, anti-atherosclerotic, anti-nephrotoxic, as well as reproprotective and hepatoprotective effects [18,21,55]. Gossypetin has been shown to exhibit therapeutic effects at doses as low as 10 mg/kg and 20 mg/kg in vivo [20,64]. Therefore, gossypetin may serve as a potential candidate in the treatment of diabetes and its associated complications.

### 1.3. Pharmacological Action of Gossypetin

**Figure 6:**
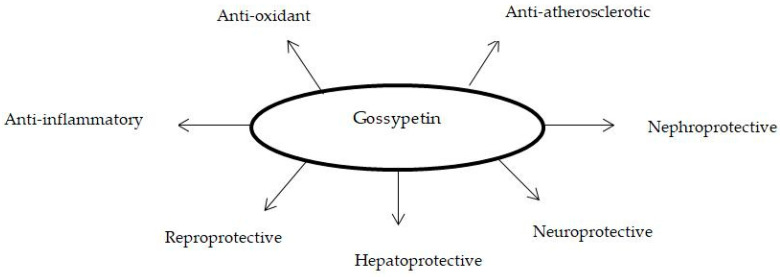
The reported pharmacological importance of gossypetin [18,19,62,66,67].

As mentioned above, diabetes mellitus causes numerous micro- and macrovascular complications in affected patients [5,65]. Gossypetin may serve as an appropriate candidate for the prevention and treatment of diabetes mellitus complications. In the below section, we discuss the therapeutic potential of gossypetin for the treatment of inflammation, oxidative stress, kidney dysfunction, liver dysfunction, cognitive dysfunction, atherosclerosis and reproductive dysfunction (Figure 6) [18,19,62,66,67].

#### 1.3.1. Anti-Oxidant and Anti-Inflammatory Effects of Gossypetin

**Figure 7:**
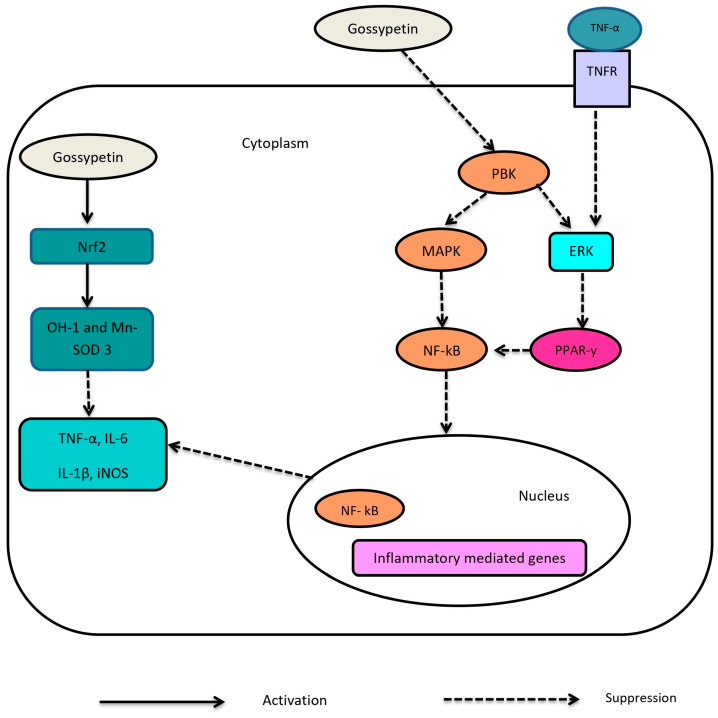
The potential molecular mechanism of gossypetin on the NF-kB inflammatory pathway [55,73]. It is shown that gossypetin inhibits PDZ-binding kinase (PBK) phosphorylation, which is involved in the regulation of p38 MAPK and ERK1/2 kinases [73]. Consequently, MAPK and ERK are inhibited by gossypetin [55,73]. The downregulation of MAPK inhibits NF-kB activation and translocation into the nucleus [55,73]. As a result, there is a downregulation in the transcription of inflammatory mediators. In addition, the downregulation of ERK inhibits PPAR-y, thereby antagonising NF-kB [55,73]. Furthermore, gossypetin has been shown to stimulate Nrf2, thereby activating OH-1 and superoxide dismutase (SOD)-3 [73]. This promotes anti-oxidant and anti-inflammatory properties.

The oxidative stress caused by ROS and free radicals has been shown to be linked with numerous complications in T2DM [68]. The cellular alterations that result in diabetic complications have been demonstrated to be caused by increased oxidative stress and inflammation, which are both a cause and an effect of diabetes [68]. Radiation promotes the production of hydroxyl radicals, which leads to lipid peroxidation and significant biological damage [69]. It is responsible for the development of inflammation through nitric acid (NO) production [69]. In a previous study, hepatocytes were treated with 20 mM and 40 mM GTIN for one hour before exposure to 5 Gy radiation [55]. The treatment with 20 mM of GTIN offered more effective protection against damage to supercoiled DNA than 40 mM of quercetin after exposure to 5 Gy radiation [70]. According to the study, GTIN significantly protected against radiation-induced DNA damage and inflammation directly and indirectly by scavenging ROS and NO [55]. The study demonstrated the anti-oxidant potential of GTIN, evidenced by the high ferric-reducing anti-oxidant power (FRAP) value [55]. FRAP denotes the total anti-oxidant pool [71]. GTIN contains a pair of parahydroxyls at positions 5 and 8 and two pairs of orthohydroxyls [55]. It is therefore oxidisable because it can form two o-quinone intermediates and one p-quinone intermediate, which aids in scavenging free radicals [55]. Moreover, it was found that GTIN inhibited free-radical-mediated DNA strand breakage and decreased radiation-mediated oxidative stress in the study’s ex vivo model [55]. According to the study, GTIN terminates a radical chain reaction by reacting with radicals, transforming them into more stable compounds [55]. In addition, in the study’s ex vivo model, it was made evident that gossypetin exhibited the most potent anti-oxidant activity at a dosage of 50 mM [70]. It was also suggested that exposure to 50 mM gossypetin exhibits therapeutic effects against DNA damage at even a higher radiation dose exposure between 5 Gy and 20 Gy [55]. The aforementioned studies have demonstrated the anti-inflammatory and anti-oxidant properties of gossypetin in the management of radiation-induced oxidative stress (Figure 7) [55,70,72,73]. Taken together, gossypetin may alleviate oxidative-stress-induced diabetic complications due to its potent anti-oxidant and anti-inflammatory potential.

#### 1.3.2. Anti-Atherosclerotic Effects of Gossypetin

One of the main complications of DM is atherosclerosis, which is caused by endothelial dysfunction, inflammation and oxidative stress [74]. According to previous studies, increased low-density lipoprotein (LDL) oxidation has been linked to hyperinsulinaemia and impaired glucose tolerance in T2DM [75,76]. Oxidised low-density lipoprotein (ox-LDL) induces damage to the vascular endothelial cells, which is a major factor in the development of atherosclerosis [77]. In T2DM, oxidative stress promotes the onset of vascular smooth muscle (VSMC) dysfunction [78]. Plaque formation results from VSMCs’ progressive proliferation and migration from the vascular media to the neointima during the pathogenesis of atherosclerosis [78]. According to a previous study, starved rat aortic VSMCs were treated with 0, 1, 5, 10, 25, 50, 100 and 250 μM GTIN for 48 h [66]. The research findings indicated that GTIN inhibits the dysfunction of VSMCs through various mechanisms, including the suppression of cell proliferation, cell cycle progression, migration, matrix degradation and oxidative stress [66]. It was concluded that GTIN may demonstrate protective effects against VSMC dysfunction, which may delay atherosclerotic pathogenesis [20,66]. According to another study, gossypetin demonstrated protective effects against endothelial damage both in vitro and in vivo [20]. In the in vitro model, cells were treated with 0.1, 0.5, 1.0, 2.0 and 5.0 μM for 24 h [20]. In the study’s in vivo model, New Zealand white rabbits were fed on a high-fat diet for 10 weeks to induce atherosclerosis and treated with 10 mg/kg gossypetin [20]. The results showed that in the in vitro model, gossypetin inhibited ox-LDL uptake, lipid-laden foam cell formation and promoted cholesterol efflux in a dose-dependent manner [20]. Furthermore, treatment with gossypetin has been shown to decrease liver injury markers and improve the lipid profile [20]. It was stated that numerous signals may have been involved in this process and the mechanisms are likely to be complex [20]. Gossypetin may have a major role in atheroprotection through upregulating autophagy [20]. Gossypetin inhibits oxidised low-density lipoprotein (ox-LDL) by activating peroxisome proliferator-activated receptor (PPAR)-α and inhibiting PPAR-γ, which promotes macrophage cholesterol clearance and delays atherosclerosis [20]. These studies have highlighted the anti-atherosclerotic property of gossypetin in the treatment of endothelial dysfunction [20,66]. Taken together, gossypetin may alleviate atherosclerosis in T2DM due to its anti-oxidant potential and ability to target endothelial dysfunction.

#### 1.3.3. Nephroprotective Effects of Gossypetin

Oxidative stress and inflammation play a major role in the development of renal dysfunction in T2DM [43,79]. A previous study has demonstrated that in a nephrotoxicity model, kidney function was significantly improved in Sprague-Dawley rats administered 30 mg/Kg GTIN intraperitoneally for 30 days [18]. Anti-oxidant enzyme activities were significantly increased by GTIN supplementation, which was also associated with a reduction in ROS and malondialdehyde (MDA) levels [18]. Higher creatinine clearance was linked to an improved glomerular filtration rate, which was reflected by a reduction in urea, creatinine, kidney injury molecule-1 (KIM-1) and neutrophil-gelatinase-associated lipocalin (NGAL) levels after GTIN supplementation [18]. Urea and creatinine are used to monitor renal function [80]. The breakdown of phosphocreatine, which is catalysed by creatine kinase, produces creatinine, which is then eliminated from the kidney via glomerular filtration [81]. Any damage to the renal tissues caused by oxidation increases the levels of urea and creatinine while decreasing creatinine clearance [81]. Additionally, urine normally does not contain the transmembrane protein KIM-1, but its presence suggests damage to the proximal tubules [81]. The results of the study showed that GTIN treatment reduced the levels of inflammatory markers such as interleukin-10 and tumour necrosis factor-α (TNF-α) [18]. The results were corroborated by previous studies which suggested that flavonoids may be responsible for suppressing inflammation [82,83]. It was suggested that the free radical scavenging and anti-inflammatory properties of GTIN may be directly related to its renoprotective effects [18]. Taken together, gossypetin may serve as a novel compound in the treatment of diabetic nephropathy due to its ability to decrease the levels of renal injury biomarkers, oxidative markers, apoptotic markers and inflammatory markers and alleviate damage to the architecture of renal tissue. In addition, the anti-oxidant and anti-inflammatory properties of gossypetin may ameliorate diabetic nephropathy by targeting oxidative stress and inflammation.

#### 1.3.4. Neuroprotective Effects of Gossypetin

Cognitive function is compromised by the oxidative stress and inflammation caused by the high blood glucose concentrations in the brain as a result of T2DM [84,85]. In a previous study, stress was used to induce memory and spatial learning deficits in mice [64]. Furthermore, long-term unpredictable stress leads to adverse consequences, such as neuropsychiatric conditions including dementia, depression and anxiety [86]. In this study, five weeks of exposure to different stressors were followed by four weeks of intraperitoneal administration of gossypetin at doses of 5, 10 and 20 mg/kg [64]. The behaviour pattern significantly improved following gossypetin administration, according to the results [64]. It has been found that mice exposed to 10 and 20 mg/kg of gossypetin had significantly lower levels of corticosterone and oxidative stress [64]. The main cause of synaptic loss and cognitive deficits is oxidative stress, which is accelerated by high cortisol levels [87]. Moreover, gossypetin administration was found to increase the levels of serotonin, norepinephrine and brain-derived neurotrophic factor (BDNF) [64]. A crucial molecule in the plastic changes linked to memory and learning is known as BDNF [88]. In patients with T2DM, lower levels of BDNF have been linked to cognitive impairment [89,90]. Another study found that at doses of 5 and 20 mg/kg po, respectively, gossypetin demonstrates significant anti-anxiety and anti-depressant activity in mice [91]. Diabetes promotes an increase in the renin–angiotensin–aldosterone system (RAAS) both locally and systemically in the brain [92]. Pro-inflammatory processes are induced in the brain by elevated RAAS activation [93]. One factor influencing the decreased synthesis and release of BDNF is neuroinflammation [94]. Reduced BDNF levels have been linked to the development of depression by promoting neuronal damage [95]. Furthermore, a previous study showed that gossypetin administration in vitro and in vivo decreased several of the hallmarks associated with Alzheimer’s disease (AD), including microgliosis and astrogliosis [67]. In the study’s in vitro model, the primary microglial cells were treated with 25 μM gossypetin for 24 h [67], and mice were intragastrically administered 10 mg/kg gossypetin daily for 13 weeks [67]. The results demonstrated that gossypetin reduced the formation of various types of beta-amyloid (Aβ) protein plaques, which delayed the progression of AD [67]. Interestingly, cognition and memory function was reversed to the same level as the control group after treatment with gossypetin [67]. Insulin resistance contributes to the development of Alzheimer’s disease by promoting the build-up of Aβ proteins [96,97]. Taken together, gossypetin may serve as a novel compound in the treatment of cognition impairments in T2DM due to its ability to reduce oxidative stress and inflammation, decrease cortisol levels and increase BDNF levels.

#### 1.3.5. Hepatoprotective Effects of Gossypetin

Systemic lipid and glucose homeostasis is maintained largely in part by the liver [98]. Studies have demonstrated that non-alcoholic fatty liver disease (NAFLD), fibrosis, cirrhosis and aberrant glycogen deposition are all associated with T2DM [99,100]. It has been demonstrated that oxidative stress and inflammation play a significant role in the development of liver dysfunction in T2DM patients [100,101]. Previous studies on drug development for nonalcoholic steatohepatitis (NASH) were based on the one-hit hypothesis [21,102]. However, the development of therapeutic agents that target one specific mechanism to improve NASH has failed [102,103]. As a result, the “multiple-hit hypothesis”, which takes into account focusing on multiple factors concurrently to improve the effectiveness of therapeutic agents, has become the new paradigm in NASH drug development [21,104]. It has been suggested that targeting oxidative stress and metabolic dysfunction may be an approach to prevent NASH since these factors promote the development of fibrosis and inflammation [105]. According to a previous study, gossypetin reduced hepatic steatosis, lobular inflammation and liver fibrosis in mice induced with NASH for four weeks at a dose of 20 mg/kg/day [21]. According to the study, gossypetin directly acted as an anti-oxidant in both the in vitro and in vivo models, reducing the oxidative stress caused by hydrogen peroxide and palmitate [21]. It supports previous studies which have shown that anti-oxidants have the ability to alleviate NASH [21,106]. Furthermore, the study revealed that gossypetin induced higher AMPK phosphorylation compared to the other flavonoids, including quercetin [21] Acetyl-CoA carboxylase 1 (ACC), an essential enzyme for fatty acid synthesis that controls hepatic insulin resistance, is phosphorylated by AMPK [21]. According to the study, mice treated with gossypetin exhibited increased AMPK activation, which reduced liver steatosis [21]. Taken together, gossypetin may serve as a novel therapeutic compound in the treatment of NASH and NAFLD due to its double targeting action against oxidative stress and AMPK phosphorylation.

#### 1.3.6. Reproprotective Effects of Gossypetin

Male reproductive dysfunction in T2DM has been demonstrated to be exacerbated by oxidative stress, which promotes changes in hormone levels, apoptosis in germ cells and a decline in semen quality [107,108]. An organic substance known as paraquat (PQ) has been shown to promote major damage to the male reproductive system [19,109]. In a previous study, male reproductive dysfunction was induced by PQ exposure through the production of free radicals [19]. In the study, adult male Sprague-Dawley rats were administered with gossypetin at doses of 5 mg/kg and 30 mg/kg for 56 days [19]. Gossypetin was found to improve PQ-induced reproductive dysfunctions in the study [19]. Gossypetin reduced the levels of reactive oxygen species (ROS) and malondialdehyde (MDA) while increasing the activities of glutathione reductase (GSR), catalase (CAT), superoxide dismutase (SOD) and glutathione peroxidase (GPx) [19]. Gossypetin also enhanced sperm viability, motility, as well as the quantity of hypo-osmotic tail-swelled spermatozoa and epididymal sperms [19]. In addition, it decreased sperm morphological abnormalities [19]. Gossypetin alleviated the luteinising hormone (LH) and follicle-stimulating hormone (FSH) reductions induced by PQ [19]. Additionally, gossypetin significantly increased the level of testosterone by upregulating the expression of steroidogenic enzymes, indicating that it has androgenic properties [19]. Gossypetin downregulated the gene expression of apoptotic markers such as caspase-3 and upregulated the gene expression of anti-apoptotic markers [19]. Taken together, gossypetin may serve as a novel compound in the treatment of male reproductive dysfunction in T2DM due to its anti-oxidant, androgenic and anti-apoptotic properties.

### 1.4. Potential Use of Gossypetin in Diabetes Mellitus

The above section highlights the reported findings of gossypetin in the treatment of dysfunctions in the renal, cardiovascular, hepatic, reproductive and cognition systems [20,21,67]. Many of the complications that occur in diabetes are associated with oxidative stress [110,111]. An imbalance between the generation of reactive oxygen species (ROS) and the anti-oxidant system’s capacity to neutralise these molecules results in oxidative stress [112]. Diabetes promotes oxidative stress due to a variety of mechanisms, such as increased oxygen radical production from glucose auto-oxidation, the production of glycated proteins and the glycation of anti-oxidant enzymes, which limits the capacity of anti-oxidants to neutralise free radicals [7,113]. Gossypetin may prevent diabetic complications due to its anti-oxidant, anti-apoptotic and anti-inflammatory actions [21,73]. Elevated blood glucose levels pose a challenge in the treatment of diabetes mellitus [114]. Consequently, therapies that lower blood glucose levels may help treat diabetes mellitus [114]. Previous studies have shown that diabetes is associated with the impairment of AMPK activity [115,116]. AMPK stimulates β-oxidation and fatty acid uptake while suppressing the synthesis of triglycerides, cholesterol and fatty acids [117]. Moreover, AMPK signaling reduces oxidative stress, inflammation and apoptosis [118]. Insulin resistance is inhibited and beta (β)-cell survival is promoted by AMPK activation [119]. AMPK activation has been shown in numerous studies to improve glucose uptake into the cells and reduce intracellular glucose production in diabetics [116,117]. In addition, metformin and rosiglitazone, the two main conventional medications used in diabetes, demonstrate their metabolic effects partially by activating AMPK [120]. This makes the pharmacological activation of AMPK a viable target for DM drug development and discovery [121]. Unfortunately, there are negative side effects associated with these conventional anti-diabetic drugs [122,123]. Consequently, it is imperative to investigate natural medications derived from plants that treat diabetes and lack adverse side effects [124]. There is significant potential for managing diabetes mellitus and its complications with fewer side effects when natural products such as quercetin are used to activate and regulate the AMPK pathway [116,125]. It is well known that gossypetin is structurally similar to quercetin with an extra hydroxyl [20,126]. Therefore, gossypetin may exhibit similar anti-diabetic properties to quercetin. Interestingly, gossypetin has been shown to possess higher AMPK activity than quercetin [21]. For this reason, gossypetin may serve as a promising treatment for diabetes mellitus and its associated complications.

### 1.5. Conclusions

In this review, we highlighted the pharmacological properties of gossypetin and its potential in the treatment of diabetes and the associated complications. Our review of the literature demonstrated that gossypetin exhibited anti-oxidant, anti-inflammatory, nephroprotective, neuroprotective and hepatoprotective properties [21,67,126]. Gossypetin ameliorates many complications by targeting oxidative stress and inflammation [17,21]. Oxidative stress and inflammation play a critical role in the development of T2DM [68]. It is worthy to note that gossypetin offered better protection against DNA damage from oxidative stress than quercetin [70]. In addition, gossypetin possessed the highest AMPK activity in comparison to other flavonoids, including quercetin [21]. Consequently, gossypetin may serve as a potential candidate in the treatment of diabetes and its associated complications. Future studies should investigate the therapeutic potential of gossypetin in diabetes mellitus. Moreover, further studies are also required to establish the pharmacokinetic and safety profiles of gossypetin.

## Data Availability

No new data were created or analysed in this study. Data sharing is not applicable to this article.
